# Androgen Action in the Ovary

**DOI:** 10.3389/fendo.2018.00452

**Published:** 2018-08-10

**Authors:** Stephen Franks, Kate Hardy

**Affiliations:** Institute of Reproductive and Developmental Biology, Imperial College London, Hammersmith Hospital, London, United Kingdom

**Keywords:** androgen receptor, follicle development, apoptosis, polycystic ovary syndrome, genomic and non-genomic actions, follicle atresia

## Abstract

Androgen production by the ovary is an essential requirement for normal cyclical secretion of estradiol but its physiological role extends to important actions on both preantral and antral follicle development, including promotion of granulosa cell proliferation. It is likely only in mature antral follicles that androgens encourage apoptosis and consequent follicle atresia, and this may be an important mechanism to ensure mono-follicular ovulation in primates, including humans. Recent studies have provided new insight into the mechanism of androgen signaling in the ovary which involves both genomic and non-genomic effects that are complementary in effecting a cellular response. In polycystic ovary syndrome, a condition characterized by intra-ovarian androgen excess, aberrant development of both preantral and antral follicles is a salient feature. We present evidence that local action of androgens plays a part in such abnormalities. Finally, we review the role of androgens in follicle atresia and conclude that the effects are part of the normal physiology of follicle maturation.

## Introduction

Whilst it is well recognized that androgens are an essential substrate for estradiol production by the ovary, the perception persists that androgens have an adverse effect on ovarian follicular development, even under physiological conditions but especially in an environment of androgen excess. This review will focus on the variety of androgen action on normal ovarian function and on the role of androgen excess in the etiology and ovarian manifestations of polycystic ovary syndrome (PCOS), the commonest endocrine disorder in women of reproductive age.

## Physiology of androgen action in the ovary

### Androgens and antral follicle function

The cyclical production of estradiol depends upon the availability of androgen, as a steroid precursor and, of course, cyclical changes in gonadotrophins. Under the influence of tonic levels of LH, androgens are produced by the theca cells of antral follicles. In the human ovary, LH receptors are present in theca cells but normally only appear in granulosa cells in mature follicles greater than 10 mm in diameter (i.e., the antral follicle that is most likely to go on to ovulate) (1). FSH receptors are present exclusively in the granulosa cells. Androgens (predominantly androstenedione and testosterone) diffuse across the basal lamina of the follicle to the granulosa layer where, under the control of FSH, they are converted to estrogen by the action of CYP19 (aromatase) (2). This co-ordinated interaction of gonadotrophins within the follicle is often referred to as the 2-cell, 2-gonadotophin process (2). Androgens may also have a role in the demise of antral follicles that form part of the cohort that undergo further growth in response to the early follicular phase rise in FSH but regress in the late follicular phase as FSH levels fall (1, 3). This is a physiological mechanism that ensures that in humans (and non-human primates), mono-follicular ovulation is the rule. The ability of androgens to induce atresia in antral follicles has often been cast as a deleterious effect, particularly under conditions of androgen excess (notably PCOS) but the role of androgens may be rather more nuanced than has been described, as suggested below (see “Androgens and follicle atresia revisited”).

### Androgen receptor expression and androgen action throughout follicle development

**Figure 1:**
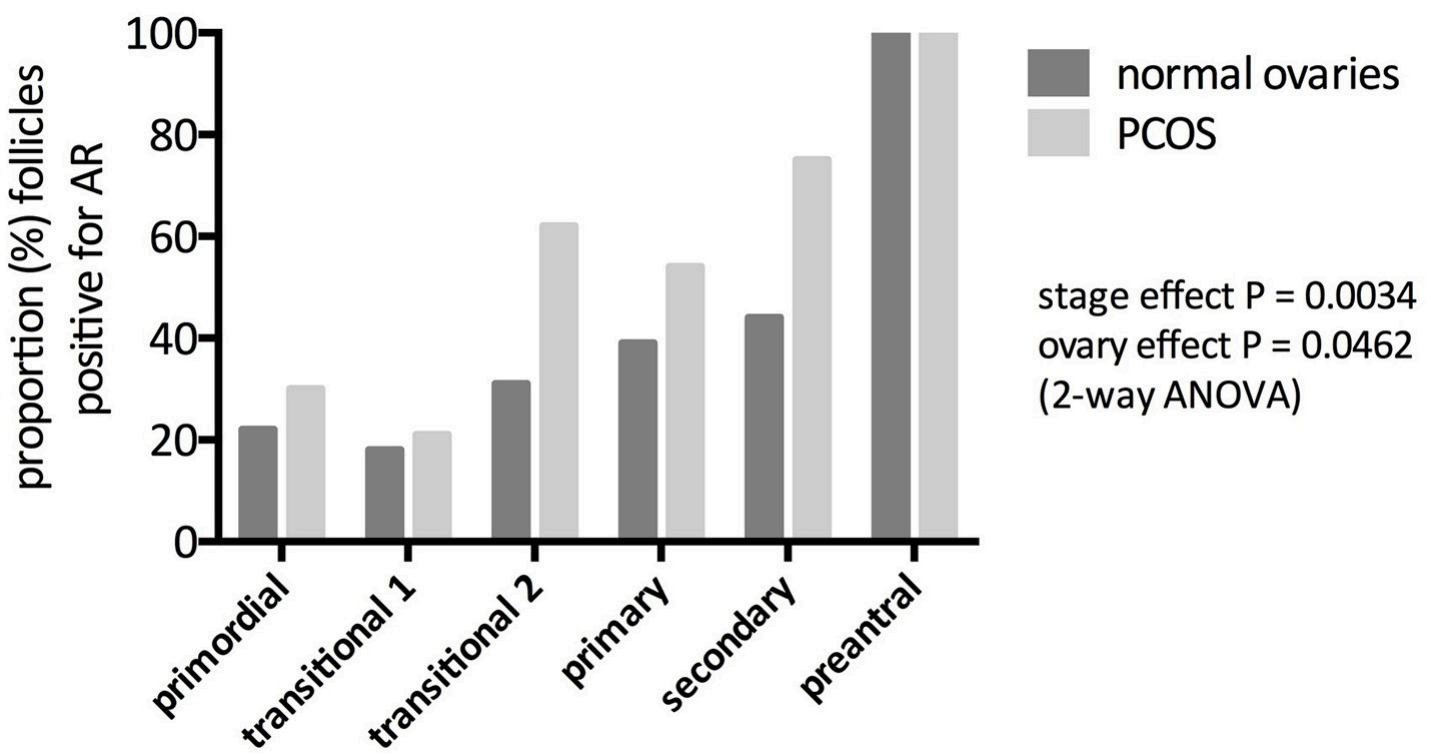
Immunohistochemical identification of androgen receptor (AR) protein in preantral follicles of women with and without PCOS. Bars represent the proportion of follicles that stain positive for AR protein. AR expression increased significantly with increasing stage of follicle development in both normal and PCOS (stage effect) but here was a significantly greater abundance of AR in PCO follicles (ovary effect). From Webber (12).

Although androgen receptor (AR) is found in all three components of the ovarian follicle, granulosa, theca and oocyte (4, 5), AR RNA and protein are most abundant in granulosa cells. In the primate ovary, there is little expression of AR in oocyte and theca of antral follicles (6) and in the human fetal ovary, AR expression is confined to somatic cells (7). Gene expression in the human ovary is high in granulosa cells of small antral follicles but reduces in pre-ovulatory follicles (8). AR expression is present in preantral follicles in rodent (9), ovine (10) as well as primate ovary, suggesting a physiological role in follicle development and function over and above the provision of substrate for estrogen production. In the human ovary, AR gene expression can be detected in human preantral follicles from the primary stage onwards (11), whilst AR protein can be observed from the primordial stage, gradually increasing during follicle development so that 100% of multi-layered, preantral follicles express AR protein (12) (Figure 1). AR gene expression is prominent in human antral follicles but it is noteworthy that peak expression is in small antral follicles (of around 6 mm in diameter) but is much reduced in larger (about 15 mm) antral follicles and lower still in the mature, preovulatory follicle (8). These changes in the level of AR expression during the later stages of follicle development may be important in terms of androgen action on survival or loss of follicles during a normal ovulatory cycle (see section on “Androgens and follicle atresia revisited” below).

There is plentiful evidence to show that androgens stimulate the growth of both preantral and antral follicles in various species (13–20). Androgen action appears be important for normal follicle development and function. Mice lacking AR in the ovary have impaired follicle maturation and reduced litter size (21–23). Recently, Walters and colleagues have shown that in a neurone-specific AR knockout mouse, there is significant disruption of the hypothalamic regulation of gonadotrophins, associated with abnormalities of ovarian follicular development (24). The potent androgen, dihydrotestosterone (DHT) stimulates protein expression of Ki67 (a marker of cell proliferation) in granulosa cells of mouse preantral follicles without effect on apoptosis (20). Androgen also increases responsiveness of granulosa cells to FSH in terms of both growth and expression of key genes involved in steroidogenesis (15, 20, 25, 26). These results reflect those of a seminal series of studies in primate ovary in which Bondy and colleagues demonstrated that *in vivo* exposure to androgen leads to growth of both preantral and antral follicles and was associated with increased expression of FSH receptor (FSHR) in granulosa cells (18, 27, 28). AR expression was found to be positively correlated with that of Ki67 and inversely related to apoptotic cell count (27).

**Figure 2:**
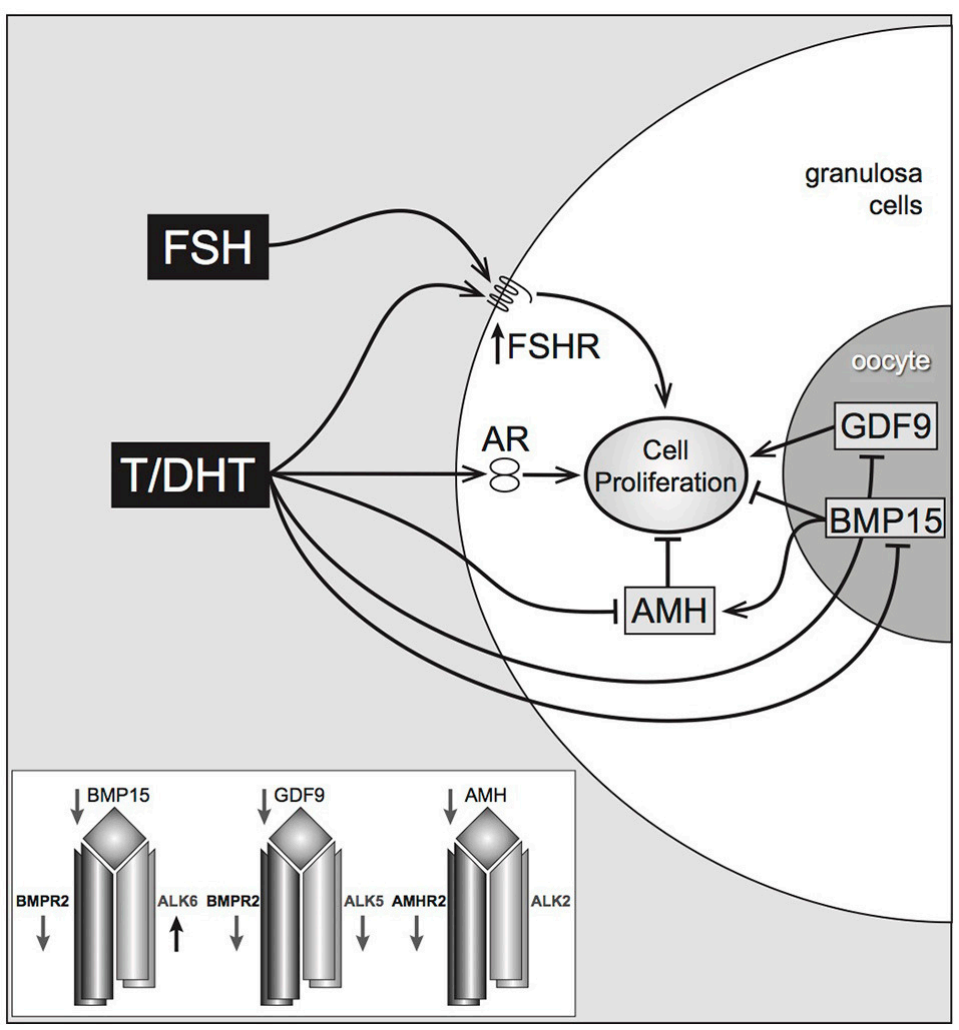
Proposed pathways of androgen action on preantral follicle growth. Testosterone or DHT act via the androgen receptor, increasing granulosa cell proliferation. This may be mediated directly, or indirectly by increased FSHR (stimulating GC proliferation) or decreased AMH (reducing AMH inhibition). AMH can be further reduced by androgen-induced reduction of oocyte-specific BMP, which normally stimulates AMH levels. Inset box summarizes androgen-induced inhibition of TGFβ ligands, and type I and II TGFβ receptors, with the exception of *Alk6* Laird et al. (20).

In isolated, mouse preantral follicles, incubation with DHT greatly enhances expression of the steroid acute regulatory protein (StAR, a key regulator of steroidogenesis) in response to FSH (20). In the same model, both testosterone and DHT interact with members of the TGFβ superfamily, most noticeably reducing gene expression of anti-Mullerian hormone (AMH) (produced by granulosa cells) and bone morphogenetic protein-15 (BMP-15) (produced by the oocyte) both of which may have inhibitory effects on follicle growth (20) (although BMP-15, particularly in the presence of GDF-9 signaling, may also have a stimulatory action on follicle growth) (29). The positive effect of DHT on FSHR expression and the negative effect on the growth-inhibitory AMH and BMP-15, suggest that DHT-stimulated growth in preantral follicles is a complex phenomenon that relies upon a balance of endocrine and local growth factor actions (Figure 2). Conversely, there is evidence that BMPs 4, 6, and 7 have inhibitory actions on androgen production by bovine theca (30).

### Androgen signaling in the ovary

The classic mode of androgen action, as for most steroids, involves binding of androgen to AR in the cell cytoplasm and translocation of the hormone-receptor complex to the nucleus where it binds to a specific sequence in the promotor of the relevant target gene and promotes gene transcription (31, 32). Whilst there is clear evidence that this pathway is operational in both physiological and pathological actions of androgens, recent work suggests that the pathway(s) of androgen signaling are more complex and involve rapid effects that do not involve classic nuclear receptor action on transcription ie non-genomic actions (22, 33, 34). These non-genomic actions have been highlighted in the work of Sen and colleagues who describe transactivation of the epidermal growth factor receptor (EGFR) by androgen. They show that androgens can activate the MAPK kinase pathway (by phosphorylation of ERK), which, classically, transduces rapid growth factor signaling (33, 35–37). In this sense, androgens appear to have growth factor properties. The action of androgens on ERK activation appear to be mediated by matrix metalloproteases (MMPs) and by paxillin (PXN), an adaptor protein which is also implicated in translocation of AR to the nucleus (22, 36). In this way, it is proposed that genomic and non-genomic actions of androgens can be co-ordinated and may work in concert (37). PXN is able to induce expression of the microRNA miR-125b which has an anti-apoptotic effect, hence promoting androgen induced follicle survival (33).

The important finding of an interaction of androgen with the EGFR is supported by data from our own laboratory. Exposure of mouse preantral follicles to a combination of DHT and EGF in culture results in stimulation of growth that is greater than either treatment alone. Furthermore, incubation of follicles with DHT in the presence of an EGF receptor inhibitor results in attenuation of the growth-promoting effect of DHT, strongly suggesting that the effect of androgen on proliferation of granulosa cells is, in part, mediated by activation of EGFR (38).

The EGF signaling pathway is one of several growth factor-signaling pathways with which androgens may interact. As previously mentioned, DHT influences expression of oocyte- and granulosa cell-derived growth factors of the TGFβ superfamily (20). Androgen can enhance both synthesis and action of insulin-like growth factors (IGFs) (18, 28). IGFs signal primarily though the PI3Kinase (PI3K) pathway and recently, Sen and colleagues have provided evidence that androgens also directly activate the PI3K signaling pathway, leading to a complex cascade of events that involve, in an initial and rapid effect, phosphorylation, and thereby inhibition, of the polycomb group protein enhancer of zeste homolog 2 (Ezh2) (34). Ezh2 appears to be a factor in regulation of LH action in the preovulatory follicle. In the longer term (24–48 h) the micro RNA miR-101 is induced which, in turn, greatly reduces the expression of Ezh2 protein (34). These findings give further support to their hypothesis that androgen action is likely to involve both genomic and non-genomic events that are closely co-ordinated.

## Androgens and polycystic ovary syndrome

### Increased androgen production in PCOS

Polycystic ovary syndrome (PCOS) is the commonest endocrine disorder in women of reproductive age (39, 40). Although there is a wide spectrum of clinical presentation, it is typically characterized by infrequent or absent ovulation together with clinical and/or biochemical evidence of androgen excess. The biochemical hall mark of PCOS is excess androgen production, predominantly of ovarian origin (39, 41).

### Androgen action in polycystic ovaries

The systemic effects of androgen excess include cutaneous manifestations (hirsutism, acne, androgenetic alopecia) and predisposition to metabolic derangement (including increased risk of type 2 diabetes mellitus) (42–49). But there are also local actions within the ovary that are characteristic of PCOS. Anovulation in PCOS is distinguished by arrest of growth of antral follicles at 5–8 mm (50). The mechanism of follicle arrest is complex but is likely to be due to the abnormal endocrine environment that includes excessive secretion of LH, insulin and androgens, all of which may contribute to premature arrest of follicles (1, 50, 51).

There is, in addition, clear evidence for aberrant development of preantral follicles in the ovaries of women with PCOS. The density of preantral follicles is increased compared with that in normal ovaries and there is a higher proportion of primordial follicles that have been activated and have started to grow (52) with evidence for accumulation (“stockpiling” at the primary stage (53). Small preantral follicles in PCOS show higher expression of the proliferation marker, mini-chromosome maintenance protein-2 (MCM-2) than that in size matched follicles of normal ovaries (54) and demonstrate prolonged survival in culture (55). These changes in early follicle development can be attributed, at least in part, to the effects of androgen. As described above, androgens stimulate preantral follicle growth in mice, rats, sheep, cows and primates (16–18, 20, 56–58). In the prenatally androgenised sheep, histological examination of ovarian cortical tissue reveals an increase in the proportion of growing preantral follicles and a reciprocal reduction in the proportion of primordial follicles, a pattern that mimics that seen in human cortical tissue in women with PCOS (52, 58).

An interesting question is whether the androgen-growth factor interactions, referred to above, play a part in aberrant early follicle development in PCOS. In that context, it has been shown that both gene and protein expression of the type 1 IGF receptor is increased in preantral follicles of women with PCOS (59). Furthermore, there are differences between normal and polycystic ovaries in growth responsiveness to IGF1 of follicles during culture of cortical tissue which suggest that PCOS follicles have been exposed to enhanced action of IGFs *in vivo* (59).

It remains unclear whether these aberrations in early follicle development contribute to the characteristic arrest of antral follicles in PCOS but the premature appearance of LH receptors in small antral follicles may provide a clue. Androgens induce FSHR expression and the acquisition of LH receptors in the dominant follicle that is destined to ovulate is an FSH-dependent event. As yet, we know little about FSHR expression in follicles of women with PCOS but it is noteworthy that cultured granulosa cells from small antral follicles in polycystic ovaries are hyper-responsive to FSH in terms of estradiol production (60, 61).

#### Androgen and the developmental origins of PCOS

The impact of excess androgen extends beyond the systemic and local effects described above Data from animal models of PCOS suggest that exposure to excess androgen during fetal life may play as significant part in the development of PCOS (62–67). In rodents, exposure to androgen in postnatal life can also reproduce some of reproductive and metabolic sequelae of PCOS (56, 57). Studies using large animal models (sheep, rhesus monkey) provide information that is perhaps more relevant to human PCOS, particularly as ovarian function is similar in terms of follicle development and mono-ovulatory cycles (62, 64, 66, 67). At critical stages during pregnancy, these animals are given very large doses of testosterone which are sufficient to overload the “buffering” of androgen action that occurs during normal pregnancy [elevated maternal plasma levels of sex hormone-binding globulin (SHBG) and activation of placental aromatase] that prevent the fetus being exposed to excess maternal androgen. The fetus is therefore androgenised and, in postnatal life, shows features which replicate many of the characteristics of PCOS including ovarian hyperandrogenism, infrequent or absent ovulation and metabolic dysfunction (65, 66). These findings raise the possibility that PCOS is a developmental disorder in which “programming” by excess androgen plays a key role—probably by epigenetic modification (64, 68). In human development, the source of excess androgen is unlikely to be maternal androgen (thanks to the protective effect of high, maternal, circulating levels of SHBG and placental aromatase). It is more plausible that, in human PCOS, the source of excess androgen is the ovary itself. We have postulated that the polycystic ovary is genetically predisposed to secrete excess androgen and that androgen excess is manifest, perhaps in the fetal ovary but more likely during the “mini-puberty” of infancy and/or at the onset of puberty itself (64, 68, 69). Certainly, there is strong evidence for a genetic basis of PCOS. Recent genome-wide association studies have indeed identified loci (such as DENND1A) that can be implicated in androgen production by theca cells (70–73).

Further evidence to support the notion that developmental programming by excess androgens plays a part in the origins of PCOS comes from data in women with congenital adrenal hyperplasia (CAH) due to 21-hydroxylase deficiency. Here, of course, the adrenal, rather than ovary is the source of excess androgen in fetal life and beyond. Women with a well-established diagnosis of CAH commonly (80% or more) have polycystic ovaries on ultrasound (74, 75) and, indeed, may have associated endocrine abnormalities including elevated serum levels of LH (76).

## Androgens and follicle atresia revisited

There is, as has been illustrated in this review, ample evidence to support the contention that androgens have a positive and, indeed obligatory, role in normal follicle growth and function. These phenomena call into question the widely perceived view that androgens are predominantly detrimental to normal ovarian function. Nevertheless, there is clear evidence that androgens have the ability to inhibit proliferation and promote apoptosis in mature antral follicles as, for example, shown in the rat ovary (77). These apparently paradoxical phenomena can perhaps be best explained by taking into account the stage of follicle development. In the menstrual cycle of humans and non-human primates, mono-follicular ovulation is the rule. In such cycles, a single, “dominant” follicle is selected from the cohort of perhaps 5–10 small antral follicles that are recruited by the early follicular phase rise in FSH. Thereafter, it is the follicle that is most responsive to FSH that continues on the path to ovulation whereas the subsidiary follicles are unable to progress because of the negative feedback on effect on FSH of rising circulating levels of estradiol (and inhibin) (1, 50). FSH deficiency clearly plays a role in the atresia of the smaller follicles but, in this context, intra-follicular androgen concentrations appear also to play an important role.

Hillier and colleagues demonstrated a biphasic action of androgens in the ovaries of a non-human primate, the marmoset. In small antral follicles, androgens augmented FSH action on aromatase activity whereas, in larger follicles, androgen had a clear inhibitory effect (26). In a classic study, McNatty and colleagues measured estradiol and androstenedione concentrations in a large number of individual, healthy and atretic human ovarian follicles. Androgen concentrations were similar in healthy and atretic follicles but atretic follicles were characterized by much lower levels of estradiol (3). This has been interpreted as an indication that a high androstenedione to estradiol ratio (i.e., an excess of androgen over estrogen) contributes to (if not causes) follicle atresia. However, it can also be viewed as an effect of FSH deficiency, which itself is the major reason for demise of subsidiary follicles. Nevertheless, the striking finding that AR expression, which is high in small antral follicles, is drastically reduced in the healthy, preovulatory follicle (8) points to the removal, or reduction, of a potentially deleterious effect of androgen on granulosa cell survival and function in the mature follicle.

## Summary

In this review, we have provided evidence that androgens have a clear and important physiological role in follicle development, at all stages, and in estrogen production by antral follicles. In PCOS, androgen excess may contribute to aberrant preantral and antral follicle function in PCOS although other endocrine (and paracrine) factors play a part. The role of androgens in causing follicle atresia, in a normal cycle, has probably been exaggerated. FSH deficiency is likely to be the major cause of atresia in subsidiary follicles in mono-ovulatory species but in these estrogen deficient, androgen dominated follicles, androgen action may contribute to follicle loss.

## Author contributions

All authors listed have made a substantial, direct and intellectual contribution to the work, and approved it for publication.

### Conflict of interest statement

The authors declare that the research was conducted in the absence of any commercial or financial relationships that could be construed as a potential conflict of interest.
